# Stable Superwetting Surface Prepared with Tilted Silicon Nanowires

**DOI:** 10.1007/s40820-016-0100-x

**Published:** 2016-07-06

**Authors:** Xiangman Meng, Ailin Zhou, Bo Wang, Yu Chen, Yun-Hui Tang, Hui Yan

**Affiliations:** School of Materials Science and Engineering, Beijing University of Technology, Beijing, 100124 People’s Republic of China

**Keywords:** Tilted silicon nanowires, Chemical etching, Superwettability, Stability

## Abstract

**Abstract:**

Large-scale uniform nanostructured surface with superwettability is crucial in both fundamental research and engineering applications. A facile and controllable approach was employed to fabricate a superwetting tilted silicon nanowires (TSNWs) surface through metal-assisted chemical etching and modification with low-surface-energy material. The contact angle (CA) measurements of the nanostructured surface show a large range from the superhydrophilicity (the CA approximate to 0°) to superhydrophobicity (the CA up to 160°). The surface becomes antiadhesion to water upon nanostructuring with a measured sliding angle (α) close to 0°. Moreover, the fluorinated TSNWs surface exhibits excellent stability and durability because strong chemical bonding has been formed on the surface.

**Graphical Abstract:**

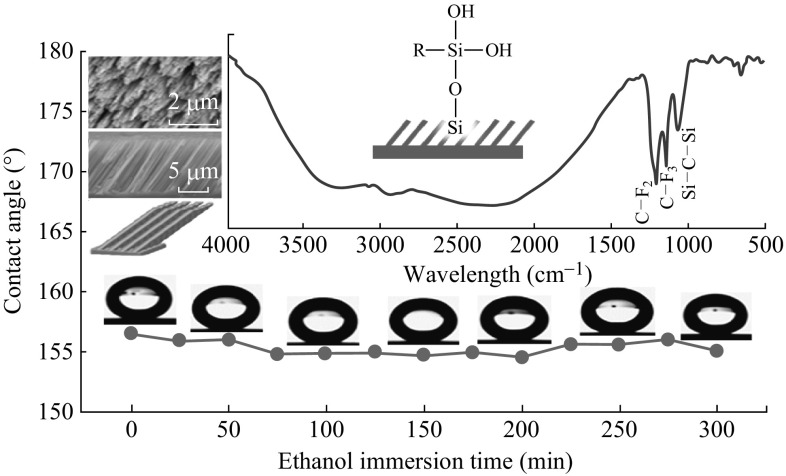

## Introduction

Superwetting surface with drop sliding angle (α) less than 5° and water contact angles (CA) lower than 5° or higher than 150° has drawn much more attention in recent years because of the limited contact angle and rollability [1, 2]. The superwetting properties of solid surface has crucial applications in our daily life as well as engineering fields, such as self-cleaning, digital microfluidic devices, biomedical engineering, and silicon hybrid solar cells [3–7]. There are two ways to obtain the superwetting properties of existing artificial surface. One is to fabricate special rough geometric microstructures on the surface, which is a successful technique to control over the wettability of solid [8, 9], the other is to modify a rough surface with low-surface-energy chemical composition [10, 11]. Recently, numerous approaches have been explored to fabricate solid surface with superwetting property, such as self-assembly, lithography selective etching of solid substrates and chemical etching [12–14], etc. However, there are still some existing limitations in the fabrication of large-scale superwetting surface and practical applications because of low stability and durability, costly and complicated fabrication procedures. Furthermore, most of the superhydrophobic surfaces have pretty high CA, chemical or geometrical heterogeneous surface which usually exhibits contact angle hysteresis and droplets adhere to the surface, so that they are greatly restricted to the applications [15].

In this study, tilted silicon nanowires (TSNWs) have been fabricated on the surface of Si subtracts by metal-assisted chemical etching (MaCE) method. The surface with TSNWs modified by material of low surface energy could greatly expand the flexible application in designing of the silicon nanowires (SiNWs) devices and may introduce new characteristics [16]. Special features of the nanostructure include a microscale smooth surface with superwetting property, antiadhesion to water, and a large range from superhydrophilicity to superhydrophobicity. Systematical investigations ranging from structural characterizations and surface wettability studies on the analysis of the mechanism have been reported. The studies are further probed into the orientation etching of SiNWs and the wettability of the silicon surface prepared with TSNWs through modified hydrophobic agent.

## Experiment

### Substrate Preparation

In our experiments, Si (110) wafers were used as etching substrates. The commercial Si wafers were cut into 2 × 1 × 0.1 cm^3^ and ultrasonically cleaned with toluene, acetone, ethanol, and deionized water (DI-water) for several cycles to entirely remove organics. To totally remove the residual metal particles of silicon surface, the cleaned Si wafers were immersed in H_2_SO_4_ (97 %) and H_2_O_2_ (40 %) oxidant solution (piranha solution) in a volume ratio 3:1 for 10 min at room temperature, followed by 1 min DI-water rinse then dried by N_2_ gas. A thin oxide layer would be formed on the surface which presents the hydrophilicity.

### Orientation Etching and Fabrication of Superwetting Surface

Before etching, the thin oxide layers were cleaned by 5 % HF solution for 3 min. The MaCE process consists of two basic steps. Firstly, placing the substrates into solution containing HF (4.8 M) and AgNO_3_ (0.01 M) for 30 s under atmosphere ambient with the purpose of silver deposition. Secondly, the Ag-loaded Si wafers were immersed in mixed solution with HF (4.8 M) and H_2_O_2_ (0.2 M) for 30 min. After the MaCE process, the as-prepared samples were dipped into dilute HNO_3_ in a volume ratio 1:1 to dissolve the silver nanoparticles (AgNPs) and then rinsed with DI-water. The etching process was controlled at 25 °C by water bath and the large-area TSNWs arrays were obtained. The nanostructured surface was then modified by a hydrophobic layer with 1H, 1H, 2H, 2H-Perfluorodecyltriethoxysilane (PFOS) and performed superwettability. The nanostructured wafers were immersed in the 1 % PFOS/ethanol solution to get the surface adopted with PFOS layer. To accelerate PFOS hydrolysis and condensation and form a stable hydrophobic nanostructured surface, the modified samples were dried at 60 °C for 5 h.

### Characterizations

The morphologies of the sample were characterized by field-emission scanning electron microscopy (FESEM, S4800) observations at 15 kV accelerating voltage. The TSNW lengths and angles were measured from the SEM images. The surface roughness (*R*a) was obtained by surface roughness measuring instrument (SU FCOM4800A) scanning multiple lines of 5 × 10^4^ μm. The FT-FIR spectrometer (Spectrum 400) was useful for the analysis of chemical composition. To characterize the wetting properties of the nanowire arrays, the CA and α measurements of water drops were performed using the Vertion 2.8 goniometer equipped from Surface Tech Co. Ltd, the droplet volumes with 5 µL water were repeated at least at five different locations on the substrate in each experiment. Self-cleaning stability was confirmed by being immersed in ethanol and outdoor test.

## Results and Discussion

### SiNWs Arrays on Silicon Wafers

**Fig. 1:**
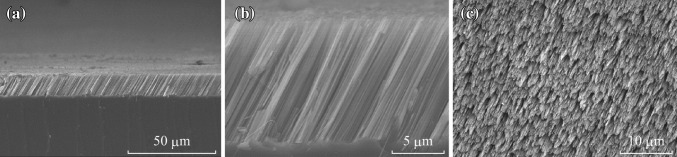
SEM images of the as-prepared SiNWs arrays on Si (110) wafer. **a** Large-scale cross-sectional SEM image, **b** magnified cross-sectional image, and **c** large-scale top view

**Fig. 2:**
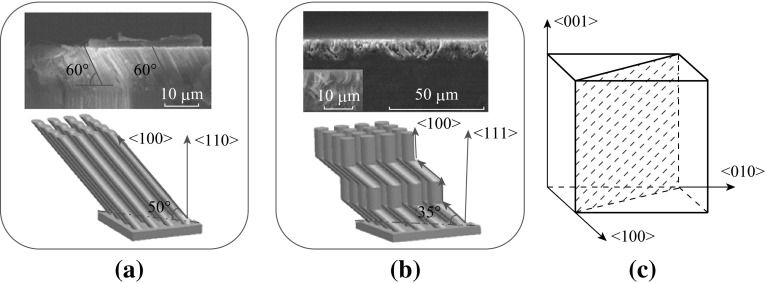
Schematic evolution of SiNWs arrays on **a** Si-(110), and **b** Si-(111) wafers. **c** Schematic of (100) directions on silicon crystal surface

The fabrication processes have been described in the above experimental section. The SEM images of as-prepared TSNWs arrays on Si-(110) wafer are shown in Fig. 1. The large-scale cross-sectional image of Fig. 1a and the top view in Fig. 1c with low magnification indicate that the SiNWs arrays obtained from Si (110) wafer are uniform on the entire wafer surface. Figure 1b shows the detailed cross section for SiNWs under high magnification in which all SiNWs are clear, long, and uniform, the length is about 13 µm, and the diameter is in the range of 80–550 nm. The TSNWs are regularly inclined to the wafer surface in both 60° on two mutually perpendicular cross sections as shown in Fig. 2a, whose direction deviates from (110) plane approximately to 50°. The direction corresponds to the crystallographic lattices of the Si (100) orientations. In another words, the etching orientation on Si (110) wafer is always along (100) direction. The etching direction can be interpreted according to lattice configuration of oriented silicon surface and the passivation effect on *H*-terminated planes. The orientation dependence mainly depends on the anisotropic dissolution of the silicon surface. The removal rate of silicon atoms from the surface in (100) direction is far faster than the other two directions. According to back-bond strength theory, the (100) plane has two bonds symmetrically directed into the reactive solution while those on the (111) plane have three, leading to a geometry that sterically prefers etching silicon atoms along the (100) direction [17, 18]. Besides, the pretreatment of HF solution in the Experimental section essentially removed the native oxide of the silicon surface, and further passivated the surface with the formation of *Si–H* terminations [19]. The *Si–H* terminations on the silicon wafer could also induce etching along (100) direction in a HF solution [20, 21].

In our experiments, one-dimensional zigzag SiNWs were obtained on Si (111) substrate, in agreement with existing reports (Fig. 2b) [7, 19]. Compared with the SiNWs prepared on Si (111), the majority of the nanowire morphologies on Si (110) wafers are more uniform. The reason is that there are three (100) directions on silicon crystal surface according to the crystal structure of silicon (Fig. 2c), and the favored (100) etching orientation always has the smallest angle relative to the substrate normal. They are nonidentically pointing outward from the surface to the internal of the Si (110) wafer, while equivalently on a Si (111) substrate (Fig. 2b). The opportunity of etching orientation along the three (100) directions are equal on Si (111) wafer, leading to a seemingly disordered nanostructure surface and the SiNWs congregated bundles. Whereas SiNWs can along the favored (100) direction on Si (110) wafers and the nanostructured surface shows uniformity [16]. The commercial silicon wafers possess an orientation deviation about ±0.5° according to GB/T12962-2005 standards, one of the two equivalent (100) directions on Si (110) wafer will be the favored etching direction, and thus the nanowire morphologies on Si (110) wafers are also uniform. Despite the variety of shapes, most SiNWs on Si substrate always oriented in the (100) direction. Based on the above experimental results, the schematic of SiNWs on three various oriented silicon wafer by MaCE method is shown in Fig. 2a, b.

### Wettability

The *R*a of TSNWs surface (0.4313 μm) is much higher than the nanostructured Si (100) wafers (0.08220 μm) and Si (111) wafers (0.1064 μm). It is well known that surface with large roughness is more conducive to the adhesion of PFOS. Thus the wettability of TSNWs surface with low-surface-energy materials was studied. The volume of water drops was set in 5 μL and the dropping height remained at 0.165 mm to avoid the effect of impaction. According to the Weber number, the impaction influence of a deposited drop on nanostructured surface is analyzed by *W* = ρ*v* ^2^ *d*/σ, in which *W* represents Weber number, ρ is water density, *ν* is impacting velocity, *d* is drop diameter, and σ is the liquid surface tension [22, 23]. In this experiment, the relevant parameters are liquid density *ρ* = 998 kg m^−3^, the drop’s impacting velocity *ν* = 0.033 m s^−1^, the drop diameter *d* = 2.6 × 10^−3^ m, and the liquid surface tension *σ* = 7.2 × 10^−3^ N m^−3^; the Weber number was about 0.039, illustrating that the effect of impaction is relatively small that can be ignored.

**Fig. 3:**
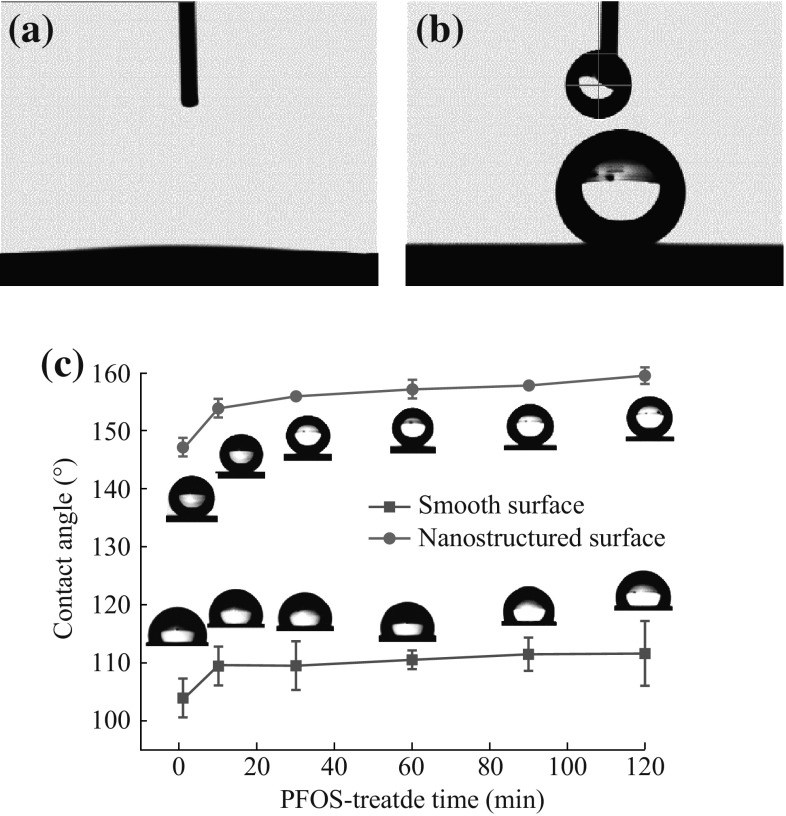
Photographs of water droplets on **a** sample S-2, **b** sample S-2 treated by PFOS. **c** Variation in the water contact angles of the smooth and nanostructured surface which be modified by PFOS against time duration

Figure 3a shows that the water droplet can nearly fully spread on the surface and the CA is less than 5°. The superhydrophilicity may be considered as a result of the *R*a and formation of some polar radicals. After being immersed in 1 % PFOS/ethanol solution for 30 min, the surface turns to be superhydrophobic and the CA approximates to 160° (Fig. 3b). The CA of the surface varied with the PFOS-treated time is shown in Fig. 3c. The CA of the smooth surface is 103° and the as-prepared surface is 147° after 1 min. When the fluorination time prolongs to 10, 30, 60, 90, and 120 min, the CA of the smooth surface is around 110° while the as-prepared surface is all above 154°. The as-prepared surface exhibits excellent superhydrophobicity. The high CA is also directly attributed to the *R*a, as mentioned above. The CA of the superhydrophobic surface is more uniform compared to nonstructured surface according to the standard error as shown in Fig. 3c. The PFOS is difficult to distribute evenly and agglomerates on the polished surface because of smoothness, while the TSNWs surface is rougher which enables the molecules to attach to the surface uniformly.

**Fig. 4:**
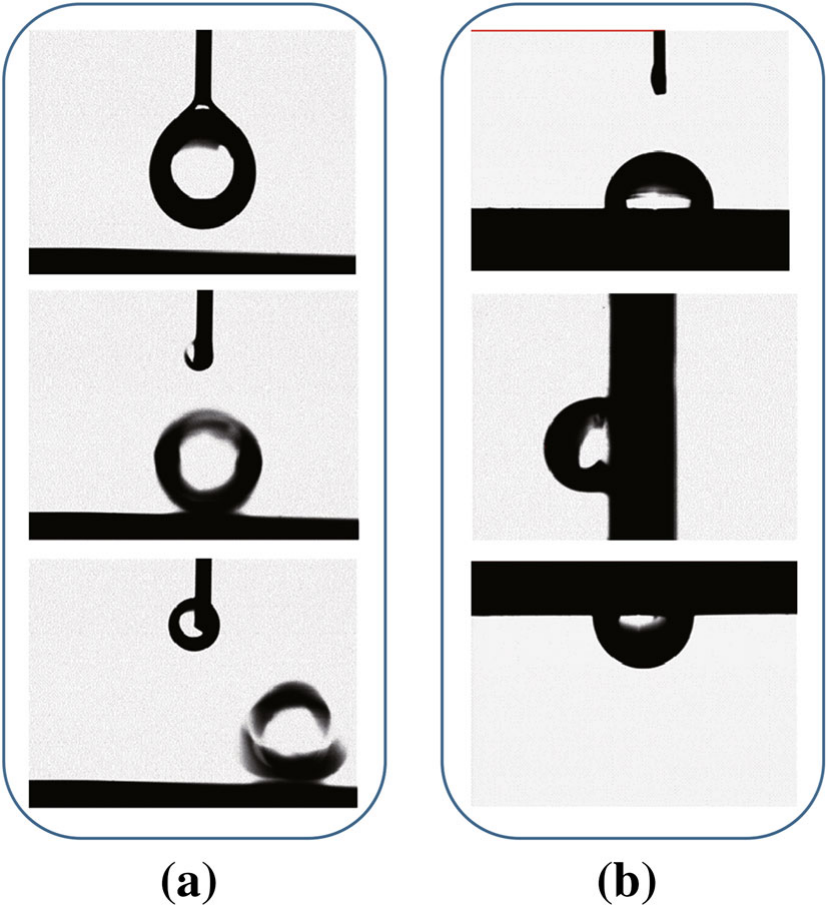
Behaviors of drops on the inclined surface. **a** Water drops on the superhydrophobic surface. **b** Water drops on PFOSS-treated Si (110) wafer

Drops deposited on the superhydrophobic surface and its sidling process with a fixed angle of 1.5° is shown in Fig. 4a. The drop slides on the surface quite quickly within short time. The surface like the lotus leaf surface have both a large CA (higher than 150°) and a small α (less than 5°), also known to be antiadhesive to water, are crucial for the superwetting surface [24]. The α on the lotus-like TSNWs surface was much lower than that on polished wafers with no nanostructures. Water droplets adhere to the smooth surface even when the wafer is tilted vertically (the pinning position, Fig. 4b, middle) or inverted (Fig. 4b, bottle). Water droplets on the water repellent surface favor rolling like an elastic ball, leaving the surface completely and keep dry or even take away the contaminants on the surface [25, 26].

**Fig. 5:**
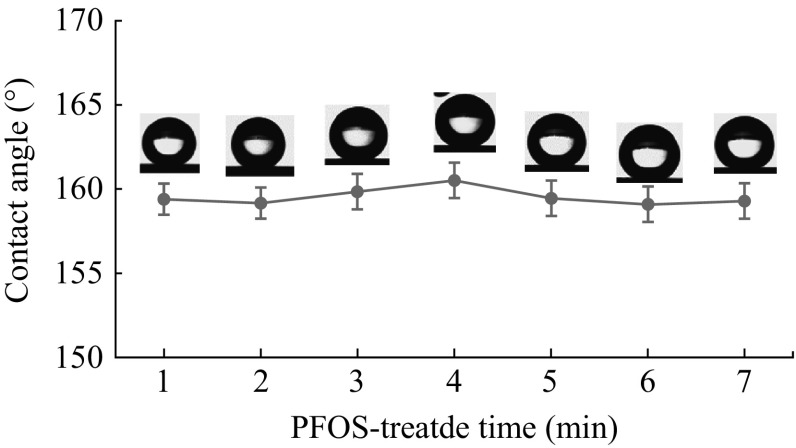
Variation of the CA against air exposure time duration. Samples were collected every day and measured for CA

**Fig. 6:**
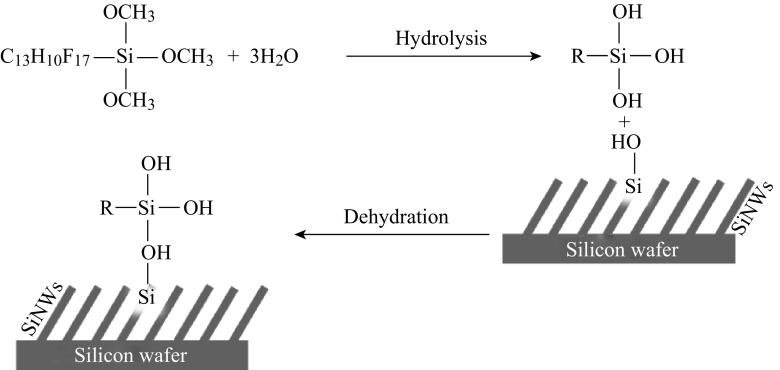
Schematic illustration of the reaction between PFOS and the substrate

Wetting stability of the Superhydrophobic nanostructured surface was also extensively studied. Figure 5 shows the CA of samples which exposed to air for 7 days, measured every day. The superhydrophobicity seems to contribute to keeping the surface clean, it shows an almost flat CA trend over a period of days. It illustrates that the PFOS attached to the silicon surface firmly and steadily. Despite the fog and haze on third days and fourth days, the CA of surface is still approximately at 159°. According to the Arkles theory [27], the hydrolysis reaction of siloxane has been formed once PFOS contacts with silicon. Through the dehydration reaction of the fluorine-bearing organosilane molecules with hydroxyl groups of SiNWs, the highly polar hydroxyl of silicon surface converts to a low polarity ether bond. Eventually, PFOS is covalently attached onto the nanostructured silicon surface and form interfacial region. Silicon surface is covered by hydrophobic film with -CF_2_ and -CF_3_ group. The surface energy of the silicon is obviously reduced and the CA has improved. The specific reaction process is shown in Fig. 6.

**Fig. 7:**
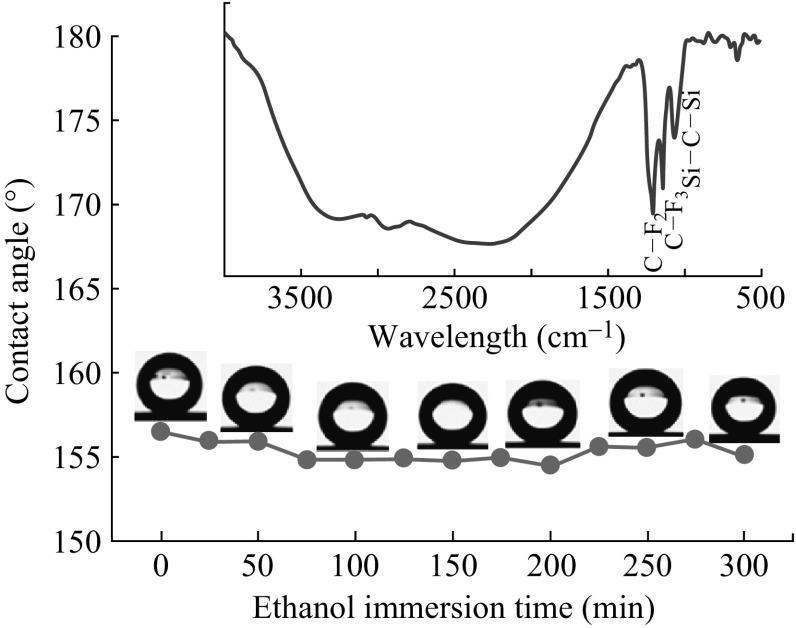
Variation of the CA against ethanol immersion time duration. The *inset* is FTIR spectrum of the superhydrophobic surface after immersed in ethanol for 300 min

After being in ethanol for a period of time, the surface shows less decrease in contact angle (Fig. 7). The inset (FTIR spectrum) shows that silane groups still exist on the silicon surface after 30 min immersion. The absorption constant at 1148 and 1200 cm^−1^ are corresponded to symmetric stretching vibration modes of -CF_3_ and asymmetric stretching vibration modes of -CF_2_, respectively. Si–O–Si asymmetric stretching vibration bands occurred at 1075 cm^−1^ [28]. It is proved that the dependence of the PFOS on the nanostructured surface is a chemical bonding. This is consistent with the above-mentioned Arkles theory.

## Conclusions

Fabrication of TSNWs arrays with extremely uniform top surface on silicon substrates by MaCE approach has been reported in this article. The etching orientation on Si wafers always follows the (100) direction. Changing the surface structure and surface chemical modification, the wetting performance of nanostructured surface can be controlled from hydrophilicity to superhydrophilic or superhydrophobicity, from adhesion with water to antiadhesion to water. The above experiments prove that the wettability of silicon surface can be evidently enhanced via chemical etching and PFOS treatments. The wetting property of superhydrophobic film is very stable with chemical bond between PFOS and substrate, which can be potentially useful for super extensive applications, including antifouling, digital microfluidic devices, and nonwetting liquid transfer.

## References

[1] Zhang J, Seeger S (2011). Polyester materials with superwetting silicone nanofi laments for oil/water separation and selective oil absorption. Adv. Funct. Mater..

[2] Zhang P, Wang S, Wang S, Jiang L (2015). Superwetting surfaces under different media: effects of surface topography on wettability. Small.

[3] Drelich J, Chibowski E, Meng DD, Terpilowski K (2011). Hydrophilic and superhydrophilic surfaces and materials. Soft Matter.

[4] Huang Y, Sarkar DK, Chen X (2011). Fabrication of superhydrophobic surfaces on aluminum alloy via electrodeposition of copper followed by electrochemical modification. Nano-Micro Lett..

[5] Xin B, Hao J (2010). Reversibly switchable wettability. Chem. Soc. Rev..

[6] Zhang Y, Cui W, Zhu Y, Zu F, Liao L, Lee ST, Sun B (2015). High efficiency hybrid PEDOT:PSS/nanostructured silicon Schottky junction solar cells by doping-free rear contact. Energ. Environ. Sci..

[7] Ghosh R, Giri PK (2016). Efficient visible light photocatalysis and tunable photoluminescence from orientation controlled mesoporous Si nanowires. RSC Adv..

[8] Lai Y, Pan F, Xu C, Fuchs H, Chi L (2013). In situ surface-modification-induced superhydrophobic patterns with reversible wettability and adhesion. Adv. Mater..

[9] Darmanin T, Guittard F (2014). Wettability of conducting polymers: from superhydrophilicity to superoleophobicity. Prog. Polym. Sci..

[10] Coffinier Y, Piret G, Das MR, Boukherroub R (2013). Effect of surface roughness and chemical composition on the wetting properties of silicon-based substrates. C. R. Chim..

[11] Yin L, Wang Y, Ding J, Wang Q, Chen Q (2012). Water condensation on superhydrophobic aluminum surfaces with different low-surface-energy coatings. Appl. Surf. Sci..

[12] Miele E, Malerba M, Dipalo M, Rondanina E, Toma A, Angelis FD (2014). Controlling wetting and self-assembly dynamics by tailored hydrophobic and oleophobic surfaces. Adv. Mater..

[13] Zhang X, Zhang J, Ren Z, Li X, Zhang X, Zhu D, Wang T, Tian T, Yang B (2009). Morphology and wettability control of silicon cone arrays using colloidal lithography. Langmuir.

[14] Bellanger H, Darmanin T, Taffin de Givenchy E, Guittard F (2014). Chemical and physical pathways for the preparation of superoleophobic surfaces and related wetting theories. Chem. Rev..

[15] Gao L, McCarthy TJ (2006). Contact angle hysteresis explained. Langmuir.

[16] Ma J, Wen L, Dong Z, Zhang T, Wang S, Jiang L (2013). Aligned silicon nanowires with fine-tunable tilting angles by metal-assisted chemical etching on off-cut wafers. Phys. Status Solidi R.

[17] Smith R, Collins S (1992). Porous silicon formation mechanisms. J. Appl. Phys..

[18] Lehmann V (1993). The physics of macropore formation in low doped n-type silicon. J. Electrochem. Soc..

[19] Chen CY, Wong CP (2015). Unveiling the shape-diversified silicon nanowires made by HF/HNO_3_ isotropic etching with the assistance of silver. Nanoscale.

[20] Christophersen M, Carstensen J, Rönnebeck S, Jäger C, Jäger W, Föll H (2001). Crystal orientation dependence and anisotropic properties of macropore formation of p- and n-type silicon. J. Electrochem. Soc..

[21] Christophersen M, Carstensen J, Feuerhake A, Föll H (2000). Crystal orientation and electrolyte dependence for macropore nucleation and stable growth on p-type Si. Mater. Sci. Eng. B.

[22] Quéré D (2013). Leidenfrost dynamics. Annual Rev. Fluid Mech..

[23] Tsai P, Hendrix MHW, Dijkstra RRM, Shui L, Lohse D (2011). Microscopic structure influencing macroscopic splash at high Weber number. Soft Matter.

[24] Li S, Li Y, Li H, Zhang L, Zhai J (2002). Super-hydrophobic surfaces: from natural to artificial. Adv. Mater..

[25] Zhang B, Li Y, Hou B (2015). One-step electrodeposition fabrication of a superhydrophobic surface on an aluminum substrate with enhanced self-cleaning and anticorrosion properties. RSC Adv..

[26] Schutzius TM, Jung S, Maitra T, Graeber G, Kohme M, Poulikakos D (2015). Spontaneous droplet trampolining on rigid superhydrophobic surfaces. Nature.

[27] Gu J, Xiao P, Huang Y, Zhang J, Chen T (2015). Controlled functionalization of carbon nanotubes as superhydrophobic material for adjustable oil/water separation. J. Mater. Chem. A.

[28] Jeong HJ, Kim DK, Lee SB, Kwon SH, Kadono K (2001). Preparation of water-repellent glass by sol-gel process using perfluoroalkylsilane and tetraethoxysilane. J. Colloid Interf. Sci..

